# Evaluation of *Camellia sinensis* Catechins as a Swine Antimicrobial Feed Additive that does not Cause Antibiotic Resistance

**DOI:** 10.1264/jsme2.ME12137

**Published:** 2012-11-09

**Authors:** Akira Ohno, Shinichiro Kataoka, Yoshikazu Ishii, Toshiaki Terasaki, Masaaki Kiso, Mitsuyuki Okubo, Keizo Yamaguchi, Kazuhiro Tateda

**Affiliations:** 1 Department of Microbiology and Infectious Diseases, School of Medicine, Faculty of Medicine, Toho University, 5–21–16 Omori-nishi, Ota-ku, Tokyo 143–8540, Japan; 2 Animal Science Division, Tokyo Metropolitan Agriculture and Forestry Research Center, 3–8–1 Fujimicho, Tachikawa city, Tokyo 190–0013, Japan

**Keywords:** catechins, green tea leaves, antimicrobial growth promoters, animal feed additives, antibiotic cross-resistance

## Abstract

Antimicrobial growth promoters (AGPs) have been banned and phased out because their use has been linked to the emergence and spread of antibiotic-resistant pathogens; however, the ban has had a marked impact on livestock production, and feed additive alternatives to AGPs are required. We focused on green tea leaves as potential alternatives to AGPs because they contain significant amounts of polyphenol catechins, which have antivirus and antimicrobial effects. We examined cross-resistance between epigallocatechin gallate (EGCG), which is the most abundant catechin of green tea leaves, and commercially available antimicrobials in clinically problematic antimicrobial-resistant bacteria, and whether bacteria have the ability to acquire resistance by consecutive passage in sub-inhibitory concentrations of EGCG. EGCG did not display any cross-resistance with reference antimicrobials and the bacteria did not acquire EGCG resistance. Further, we examined the growth-promoting effects of dried green tea leaves on the breeding of a new Japanese breed, Tokyo-X pigs. While the mortality rates of the green tea leaf (GTL) and AGP groups were both 11.1% (one in nine piglets), the mortality rate was 50% for the control group with an additive-free diet (four in eight piglets). The rate of body weight increase in both the GTL and AGP groups was approximately the same. The growth-promoting effects of green tea leaves and AGPs were similar, and there was no possibility that the antimicrobial properties of catechins caused the same problem as AGPs. Thus, it can be concluded that green tea leaves are a safe feed additive alternative to AGPs.

The widespread use of antimicrobial growth promoters (AGPs) in food animal production has had marked benefits, including improved growth rate, better feed conversion efficiency and, in some cases, enhanced public health through the elimination of zoonotic pathogens that can cause disease in humans (19). Despite these benefits, the use of AGPs has a risk of resulting in the emergence of antibiotic-resistant bacteria that can be transmitted to humans through the food chain (22). For example, avoparcin, a glycopeptide widely used as a growth promoter in animal feeds, has led to the emergence of vancomycin-resistant enterococci (VRE) in animals, which has then spread to humans (13).

In 1986, Sweden became the first country to ban the use of all AGPs. Denmark also ended the use of avoparcin and virginiamycin in 1995 and 1998, respectively. The World Health Organization (WHO) recommended that the use of AGPs from classes used in humans should be terminated or rapidly phased out, through legislation if necessary (WHO press release, 13 Jun 2000; http://www.who.int/inf-pr-2000/en/pr2000-43.html). The European Commission ultimately decided to ban the marketing and use of AGPs in January 2006 (15).

In countries outside of Europe, consumer pressure is also leading to the cessation of AGP use (5). The banning of AGPs, however, has led to a variety of problems, including decreased animal performance, inferior feed conversion efficiency, and a rise in the incidence of certain diseases, such as porcine proliferative enteropathy (1); therefore, alternatives with effects similar to those of AGPs are desirable. Exogenous enzymes, organic acids, probiotics, prebiotics and herbs or etheric oils have been tested as alternatives to AGPs (11).

Green tea (*Camellia sinensis*) is widely drunk in many Asian countries and its leaves are rich in the flavanol group of polyphenols, also known as catechins. It has been reported that catechins account for 30–42% of the dry weight of green tea solids obtained through brewing (12). We took particular note of dried green tea leaves as potential alternatives to AGPs because catechins have a variety of pharmacological properties, such as significant antioxidant, anti-cancer, anti-inflammatory, antiviral and antibacterial effects (3, 4); however, if tea leaves were used as a feed additive, there is concern that cross-resistance would develop between catechins and well-established antimicrobials.

In this study, we performed field research using Tokyo-X pigs, a new Japanese breed of domestic pig created by combining bloodlines from the Duroc (USA), Berkshire (UK), and Beijing Black (China) breeds, to compare the beneficial effects of dried green tea leaves as an alternative feed additive with those of AGPs.

We also examined the cross-resistance of clinically problematic antibiotic-resistant bacteria between epigallocatechin gallate (EGCG), which is the most abundant and most physiologically active green tea catechin (10), and a set of antimicrobial agents. We compared the development of resistance in *Staphylococcus aureus* and *Pseudomonas aeruginosa* during continuous exposure to EGCG with the resistance developed by these microbes to other antimicrobials.

## Materials and Methods

### Chemicals

Epigallocatechin gallate (EGCG), ampicillin (AMP), cefotaxime sodium salt (CTX), ceftazidime hydrate (CAZ), minocycline hydrochloride (MIN), gentamicin sulphate (GEM), erythromycin (ERY), levofloxacin (LVX) and vancomycin hydrochloride (VAN) were purchased from Sigma-Aldrich Corporation (St. Louis, MO).

### Bacterial isolates

The following bacteria were used to determine the minimum inhibitory concentration (MIC) of the above antimicrobials: 18 isolates each of methicillin-resistant and methicillin-susceptible *Staphylococcus aureus*, 25 isolates each of macrolide-resistant and macrolide-susceptible *Streptococcus pyogenes*, 24 isolates each of penicillin-resistant and penicillin-susceptible *Streptococcus pneumoniae*, 16 isolates of fluoroquinolone-resistant *Pseudomonas aeruginosa* and 14 isolates of fluoroquinolone-susceptible *P. aeruginosa*. These were isolated between late 2009 and the early part of 2010 in the clinical laboratory of Omori Medical Center, Toho University. Prof. Yasuyoshi Ike (Department Microbiology, Gunma University Faculty of Medicine) kindly provided six isolates of VanA-type glycopeptide-resistant *Enterococcus faecium* and 18 isolates each of glycopeptide-susceptible *E. faecium* and extended-spectrum beta-lactamase (ESBL)-producing *Escherichia coli* strains with fluoroquinolone resistance, and corresponding susceptible strains were provided by the Levofloxacin Surveillance Group (23). *S. aureus* FDA209P and *P. aeruginosa* ATCC 27853 were used for the *in vitro* acquired drug resistance test.

### Susceptibility testing (determination of MIC)

Antimicrobial susceptibility testing was performed using the broth microdilution method, based on the guidelines of the Clinical and Laboratory Standards Institute (CLSI) (2). EGCG and LVX were first dissolved in methyl alcohol or 0.1 mol μL^−1^ NaOH at a final concentration of 0.1% (v/v) and then prepared at the desired concentrations in cation-adjusted Mueller Hinton broth (CAMHB). Other agents were prepared in CAMHB.

The susceptibility of clinically-important antimicrobial-resistant isolates and the corresponding susceptible isolates to various antimicrobial agents were shown by the geometric mean MIC (G-MIC), and the presence or absence of cross-resistance in specific antibiotic-resistant bacteria was expressed by the R/S ratio, the ratio of the G-MIC value of susceptible to resistant isolates, and a ratio ≤1.0 indicates an absence of cross-resistance.

### Antibiotic resistance acquisition test by in vitro serial passage in sub-MIC

First, the MICs of EGCG and other antimicrobials against *S. aureus* FDA 209P and *P. aeruginosa* ATCC 27853 were determined by the broth microdilution method using 96-well microplates. Twenty-four hours later, the bacterial populations grown in each 1/2 MIC or 1/4 MIC were transferred into 0.5 mL Eppendorf tubes. Each tube was centrifuged at 15,000 rpm, washed twice with sterile saline and adjusted to MacFarland 0.5. Next, 5 μL bacterial suspension was inoculated into a freshly prepared MIC test plate. This was repeated 20 times, and the stepwise elevation in the MIC value was recorded as 2^n^ to the first MIC value instead of the MIC value. A result within 2^±1^ of the first MIC value was interpreted as an error range.

### Field studies

Animals were farmed in accordance with “Standards of Rearing Hygiene Management” for Tokyo-X pigs specified by the Tokyo metropolitan government (20). To examine the effect of green tea leaves as a food additive alternative to antibiotics, field studies were performed at the Experiment Swine Farm, Tokyo Metropolitan Agriculture and Forestry Research Center (TMAFRC). Four pigs gave birth to 26 piglets. Newborn piglets were suckled by their own mothers only, not by other mothers. They were housed together in a birthing chamber with their mother while suckling and were weaned at 4 weeks old. Weaned piglets were divided into three groups: nine pigs each in the green tea leaf (GTL) and AGP groups and eight in the control group (food additive-free), and moved into three experimental rooms with an open system. The rooms had identical conditions and ad libitum feeding was allowed by all piglets using a self-feeder. After weaning, a mixture of avilamycin (a group of oligosaccharides of the orthosomycin family) and colistin was added to the feed at a concentration of 100 mg titer g^−1^ in the AGP group. Green tea powder (Pure Green), pulverized dried leaves containing catechins of 114 mg g^−1^, was added to the feed at a final concentration of 1% (w/w) in the GTL group. A food additive-free diet as the control diet without dietary growth promotants was given to the control group. Mortality and body weight were monitored every week after birth.

### Statistical analysis

Values are expressed as the mean±SD. Differences between groups were examined for statistical significance using the log-rank test for the mortality rate and a non-parametric test (Mann-Whitney) for body weight change. *P*<0.05 was considered significant.

## Results

### Antimicrobial susceptibility tests

The results of the antimicrobial susceptibility tests are shown by the G-MIC and R/S ratio in Table 1. The G-MICs of EGCG against MRSA and MSSA for each of the 18 isolates were 16.0 and 24.4 μg mL^−1^, respectively, and the R/S ratio of MSSA to MRSA was 0.66. The R/S ratios of other antimicrobials were 10.1 for AMP, 69.2 for ERY, 1.17 for GEM, 34.4 for MIN and 90.4 for LVX. These ratios were very high except for GEM. The MICs of EGCG against 25 isolates each of macrolide-resistant and macrolide-susceptible *S. pyogenes* were >1024 μg mL^−1^. The G-MICs of EGCG against 25 isolates each of penicillin-resistant and penicillin-susceptible *S. pneumoniae* were 837 and 699 μg mL^−1^, respectively, and the R/S ratio of PSSP to PRSP was 1.20. The R/S ratios of PSSP to PRSP for AMP, CTX, MIN, ERY and LVX were 16.3, 5.94, 1.71, 0.55 and 0.90, respectively. The G-MICs of EGCG against eight isolates of glycopeptide-resistant *E. faecium* (GRE) and six isolates of glycopeptide-susceptible *E. faecium* (GSE) were 237 and 362 μg mL^−1^, respectively, and the R/S ratio of GSE to GRE was 0.66. The R/S ratios of GSE to GRE for GM, MIN and VAN were 18.0, 12.3 and 326, respectively. The G-MIC of EGCG against 18 isolates of ESBL-producing and fluoroquinolone-resistant *E. coli* was 1340 μg mL^−1^, while the G-MIC of EGCG against 18 isolates of non-ESBL-producing *E. coli* was 2048 μg mL^−1^, and the R/S ratio of non-ESBL-producing isolates to ESBL-producing isolates was 0.65. In contrast, the R/S ratios for AMP, CTX, GEM, MIN and LVX were 34.5, 1343, 34.7, 8.30 and 256, respectively. The G-MICs of EGCG against 16 isolates of fluoroquinolone-resistant *P. aeruginosa* (QRPA) and 18 isolates of fluoroquinolone-susceptible *P. aeruginosa* (QSPA) were 565 and 304 μg mL^−1^, respectively. The R/S ratio of QSPA to QRPA was 1.86, and the ratios for GEM, MIN and LVX were 1.56, 2.00 and 39.0, respectively.

### Antibiotic resistance acquisition test by in vitro serial passage in sub-MIC

**Fig. 1:**
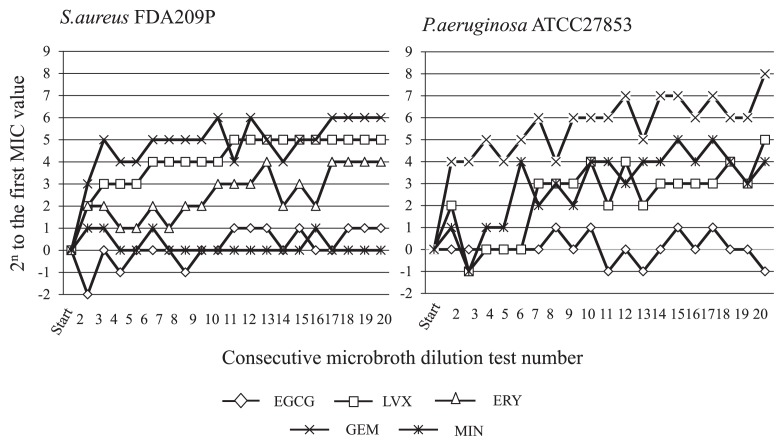
Increased MICs of epigallocatechin gallate and various antimicrobials against *P. aeruginosa* ATCC27853 and *S. aureus* FDA 209P by 20 consecutive broth microdilution tests. EGCG, epigallocatechin gallate; LVX, levofloxacin; GEM, gentamicin; MIN, minocycline; ERY, erythromycin.

Fig. 1 shows the rate of MIC increase for EGCG and each antimicrobial against *S. aureus* FDA 209P and *P. aeruginosa* ATCC 27853 observed in 20 consecutive MIC determinations by inoculation of the bacterial population grown in the sub-MIC well. The rate of MIC increase for GEM, LVX and ERY against *S. aureus* FDA209P was markedly high; 2^6^, 2^5^ and 2^4^ at 20 passages; however, EGCG and MIN did not show any increase in MIC. No MIC increase against *P. aeruginosa* ATCC 27853 was noted for EGCG. In contrast, a rise of 2^8^, 2^5^ and 2^4^ was observed for GEM, LVX and MIN, respectively.

### Mortality rate and changes in body weight at the Experiment Swine Farm

**Fig. 2:**
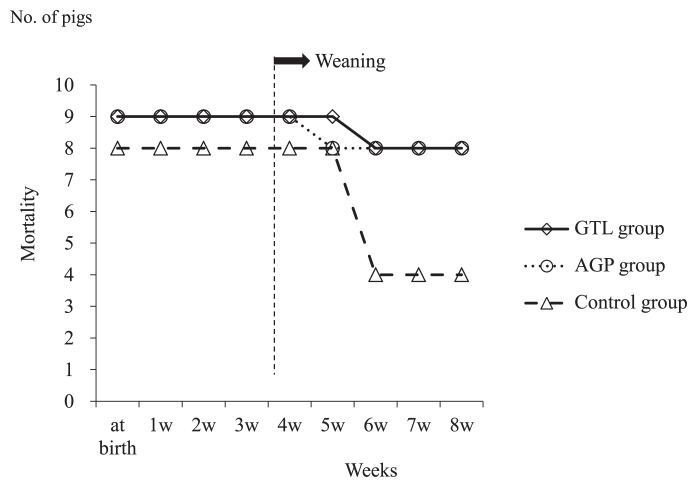
Mortality according to the feeding protocol. Pigs (n=8) fed normal feed free of food additives were the control group. GTL group was pigs (n=9) fed normal feed containing shredded dried green tea leaves at a final concentration of 1%. AGP group was pigs (n=9) fed normal feed containing avilamycin (a mixture of oligosaccharides of the orthosomycin group and colistin) at a concentration of 100 mg g^−1^.

Piglets died after weaning in all groups. While the mortality rate in both the GTL and AGP groups was 11.1% (one in nine piglets), it was 50% in the control group (four in eight piglets) (Fig. 2); however, no significant differences were shown among groups (log-rank test).

In body weight change, there was a significant difference at 1, 3, 4, 5, 6, 7 and 8 weeks after birth between the AGP and control groups, but only at 1 week after birth between the GTL and control groups. There was no significant difference between the GTL and AGP groups and subsequent growth during the study period (non-parametric Mann-Whitney test) (Table 2).

## Discussion

It is reported that withdrawal of AGPs from weaning pigs resulted in a decline in the average daily weight gain from 422 g d^−1^ in 1995 to 415 g d^−1^ in 2001 and an increase in the mortality rate from 2.7% to 3.5% during the same period (5). This *in vivo* experiment on Tokyo X pigs re-confirmed the beneficial effects of AGPs on animal growth due to reduced competition for nutrients and a reduction in microbial metabolites that are known to depress growth (7), and demonstrated that there was at least the same benefit in the GTL and AGP groups because low mortality and increased body weight were approximately the same in both groups.

In contrast, the average birth weight in the control group was a little higher than in the GLP and AGP groups and remained high in the four surviving piglets. The variability of weight among groups may have been due to a difference in litter size and milk-producing ability.

On the other hand, maternal pigs were vaccinated against atrophic rhinitis of swine, swine erysipelas and porcine circovirus infection. Periodical inspection was also conducted for Aujeszky’s disease and porcine reproductive and respiratory syndrome; therefore, the mortality rate of 50% in the control group was probably due to the considerable increase in the incidence of diarrhea 1–2 weeks after weaning (data not shown). The PCR-positive rate for group C rotavirus and bacterial numbers of a coliform group increased in individuals with diarrhea (data not shown). Taken together, diarrhea in the control group probably triggered group C rotavirus and was worsened by proliferation of the coliform group. In contrast, AGP and green tea leaves most likely prevented diarrhea through antibacterial effects and the astringency of tannin (6), one of the phenolic compounds in green tea leaves, respectively. Further, it was reported by Mukoyama *et al.* (17) that epigallocatechin gallate from green tea inhibited infection of cultured rhesus monkey kidney MA 104 cells with rotaviruses and enteroviruses; therefore, it cannot be rejected that catechins suppress rotaviruses. In addition, green tea leaves contain many trace compounds other than catechins that have physiological effects: tannin (astringency), caffeine (awakening property), theanin (stress-buffering effect), gamma-amino butyric acid (blood-pressure lowering), vitamins (effect as a coenzyme, antioxidant effect and others), saponin (blood-pressure lowering), chlorophyll and trace elements. The combined effects of these compounds may also have a growth-promoting effect in swine.

The G-MICs of EGCG against problematic clinical isolates of antibiotic-resistant bacteria such as MRSA, VRE, multidrug-resistant *P. aeruginosa* and ESBL-producing *E. coli* were nearly identical to those of the corresponding susceptible isolates, except that EGCG had little antibiotic effect against *S. pyogenes* and *S. pneumoniae*. The results indicated that EGCG displayed no cross-resistance to antimicrobials in these problematic antibiotic-resistant bacteria.

Catechins are a single compound having multi-functional antibacterial actions, including inhibition of bacterial DNA gyrase through interaction with its ATP binding site (8), inhibition of the FabG and FabI reductases of bacterial type II fatty-acid synthase (25), restoration of the antibacterial activity of beta-lactams (26) and synergy between catechins and beta-lactams against MRSA by the direct binding of catechins to peptidoglycan (27). Although each of these antibacterial functions in catechins is different from the known antibacterial mechanisms of commercially available drugs, the unique antibacterial mechanisms of catechins might explain, at least in part, the lack of cross-resistance with other antimicrobials. However, it is not easy to explain why catechins did not also develop resistance in the in vitro serial passage test while reference antimicrobials rapidly developed resistance.

Some recent reports stated that multidrug resistance could develop by sequential single-drug resistance steps in combination therapy with synergistic interactions that enhance the effect of treatment, while antagonistic drug combinations rather slow the evolution of resistance, although the treatment benefit wanes (9, 16). Treatment by synergistic drug combinations has long been of particular interest to clinicians. At the same time, antagonistic interactions are generally avoided. Yeh *et al.* (24) mentioned that the choice between synergy and antagonism may involve a trade-off between immediate efficacy of inhibition of microbial growth and future forestallment of the evolution of resistance. It may be difficult to prove whether multi-antibacterial functions in single compound catechins act synergistically or antagonistically; however, the finding that catechins did not allow resistance to develop easily may suggest that the development of bifunctional antimicrobials has great potential as a source of new antimicrobials that do not cause resistance.

It was reported that the amount of EGCG in dried green tea leaves varied from 14 to 48 mg g^−1^ in 14 commercial green tea samples (14). The daily dried feed requirement for piglets is 0.4 kg at 5 kg body weight and 0.7 kg at 10 kg body weight. As dried green tea leaves were added to the feed at a final concentration of 1%, the required dose of EGCG is 56–192 mg per 5 kg body weight and 98–336 mg per 10 kg body weight. However, Unno *et al.* (21) determined that the human serum level of epigallocatechin gallate 1, 2, 4 and 6 h after ingestion of one cup of tea containing 5 g green tea powder was 2.1% by dry weight. The time to maximum blood concentration was approximately 2 h after ingestion and the highest level of EGCG in serum ranged from 63 to 142 ng mL^−1^ (87±37 ng mL^−1^) in four volunteers. Apart from the relationship with the emergence of cross-resistance to well-known antimicrobials, these reports suggest that little antimicrobial activity was shown by the serum concentration of EGCG after dietary intake in the GTL group.

In conclusion, it can be concluded that the risk of the emergence of problematic antimicrobial-resistant bacteria following the use of dried green tea leaves containing high-abundance catechins as an alternative to AGPs is extremely unlikely. Also, studies in swine indicated that the growth-promoting effects of dried green tea leaves are equivalent to those of AGP and that dried green tea leaves are potentially suitable feed additives instead of antibiotics.

## Figures and Tables

**Table 1:** Comparison of geometric mean MIC value and the R/S ratio of epigallocatechin gallate and a well-known antimicrobial against problematic antimicrobial-resistant clinical isolates and their susceptible counterparts

Organism		Geometric Mean MIC (μg mL ^−1^)^b^							
	
Species	Resistant type^a^	EGCG	AMP	CTX	ERY	GEM	MIN	LVX	VAN
*S. pyogenes*	MSSP (25)	>1024	2.00	≤0.06	≤0.06	NT	0.29	0.84	NT
	MRSP (25)	>1024	2.50	0.10	17.4	NT	0.24	0.61	NT
	R/S ratio	1.00	1.25	1.62	290	—	0.81	0.73	—

*S. pneumoniae*	PSSP (24)	699	1.07	0.19	8.00	NT	2.55	0.45	NT
	PRSP (24)	837	17.4	1.12	4.36	NT	4.36	0.41	NT
	R/S ratio	1.20	16.3	5.94	0.55	—	1.71	0.90	—

*S. aureus*	MSSA (18)	24.4	1.47	NT	3.70	7.13	0.08	0.25	NT
	MRSA (18)	16.0	14.8	NT	>128	8.31	2.70	22.6	NT
	R/S ratio	0.66	10.1	—	69.2	1.17	34.4	90.4	—

*E. faecium*	GSE (6)	362	NT	NT	NT	2.83	1.00	NT	0.62
	GRE (8)	237	NT	NT	NT	50.8	12.3	NT	203
	R/S ratio	0.66	—	—	—	18.0	12.3	—	326

*E. coli*	NESBL (18)	2048	7.41	0.07	NT	0.58	0.96	0.09	NT
	ESBL (18)	1340	256	94.0	NT	20.1	8.00	21.8	NT
	R/S ratio	0.65	34.5	1343	—	34.7	8.30	256	—

*P. aeruginosa*	FQSPA (18)	304	NT	NT	NT	1.35	22.6	0.39	NT
	FQRPA (18)	565	NT	NT	NT	2.10	45.3	15.2	NT
	R/S ratio	1.86	—	—	—	1.56	2.00	39.0	—

a MSSP, macrolide-susceptible *S. pyogenes*; MRSP, macrolide-resistant *S. pyogenes*; PSSP, penicillin-susceptible *S. pneumoniae*; PRSP, penicillin-resistant *S. pneumoniae*; MSSA, methicillin-susceptible *S. aureus*; MRSA, methicillin-resistant *S. aureus*; GSE, glycopeptide-susceptible *E. faecium*; NESBL, non-extended-spectrum beta-lactamase; ESBL, extended-spectrum beta-lactamase; FQSPA, fluoroquinolone-susceptible *P. aeruginosa*; FQRPA, fluoroquinolone-resistant *P. aeruginosa*. R/S denotes the ratio of geometric mean MIC value of resistant to susceptible isolates.

b EGCG, epigallocatechin gallate; AMP, ampicillin; CTX, cefotaxime; ERY, erythromycin; GEN, gentamicin; MIN, minocycline; LVX, levofloxacin; VAN, vancomycin.

**Table 2:** Serial changes in swine body weight

Group		at birth	1 week	2 week	3 week	4 week	5 week	6 week	7 week	8 week
G (GTL group; n=9)		1.24±0.32	2.17±0.46	3.26±0.73	4.34±0.96	5.54±1.29	5.98±1.36	6.73±1.67	8.69±2.31	11.63±3.35
A (AGP group; n=9)		1.32±0.15	2.08±0.27	3.37±0.39	4.10±0.59	5.06±0.95	5.45±0.58	6.80±0.93	9.23±0.81	11.68±1.35
C (Control group; n=8)		1.40±0.21	2.60±0.28	3.71±0.48	4.85±0.71	6.30±1.20	6.86±1.04	8.40±0.49	10.80±0.84	14.18±1.38
Mann-Whitney^a^	G *vs.* A	—	—	—	—	—	—	—	—	—
	G *vs.* C	—	*	—	—	—	—	—	—	—
	A *vs.* C	—	**	—	*	*	*	*	*	*

a Statistical significance examined by the non-parametric Mann-Whitney test is shown:

* , *P*<0.05;

** , *P*<0.01;

—, not significant.
